# Safety measures for COVID-19 do not compromise the outcomes of patients undergoing primary percutaneous coronary intervention: a single center retrospective study

**DOI:** 10.1038/s41598-021-89419-6

**Published:** 2021-05-11

**Authors:** Xiaonan Guan, Jianjun Zhang, Yanbing Li, Ning Ma

**Affiliations:** Center of Cardiology, Beijing Chaoyang Hospital, Capital Medical University, 5 Jingyuan Road, Beijing, 100043 China

**Keywords:** Interventional cardiology, Cardiology, Diseases, Signs and symptoms

## Abstract

Coronavirus disease 2019 (COVID-19) is a global pandemic impacting nearly 170 countries/regions and millions of patients worldwide. Patients with acute myocardial infarction (AMI) still need to be treated at percutaneous coronary intervention (PCI) centers with relevant safety measures. This retrospective study was conducted to assess the therapeutic outcomes of PCI performed under the safety measures and normal conditions. AMI patients undergoing PCI between January 24 to April 30, 2020 were performed under safety measures for COVID-19. Patients received pulmonary computed tomography (CT) and underwent PCI in negative pressure ICU. Cardiac catheterization laboratory (CCL) staff and physicians worked with level III personal protection. Demographic and clinical data, such as door-to-balloon (DTB) time, operation time, complications for patients in this period (COVID-19 group) and the same period in 2019 (2019 group) were retrieved and analyzed. COVID-19 and 2019 groups had 37 and 96 patients, respectively. There was no significant difference in age, gender, BMI and comorbidity between the two groups. DTB time and operation time were similar between the two groups (60.0 ± 12.39 vs 58.83 ± 12.85 min, *p* = 0.636; 61.46 ± 9.91 vs 62.55 ± 10.72 min, *p* = 0.592). Hospital stay time in COVID-19 group was significantly shorter (6.78 ± 2.14 vs 8.85 ± 2.64 days, *p* < 0.001). The incidences of malignant arrhythmia and Takotsubo Syndrome in COVID-19 group were higher than 2019 group significantly (16.22% vs 5.21%, *p* = 0.039; 10.81% vs 1.04% *p* = 0.008). During hospitalization and 3-month follow-up, the incidence of major adverse cardiovascular events and mortality in the two groups were statistically similar (35.13% vs 14.58%, *p* = 0.094; 16.22% vs 8.33%, *p* = 0.184). The risk of major adverse cardiac events (MACE) was associated with cardiogenic shock (OR, 11.53; 95% CI, 2.888–46.036; p = 0.001), malignant arrhythmias (OR, 7.176; 95% CI, 1.893–27.203; p = 0.004) and advanced age (≥ 75 years) (OR, 6.718; 95% CI, 1.738–25.964; *p* = 0.006). Cardiogenic shock (OR, 17.663; 95% CI, 5.5–56.762; *p* < 0.001) and malignant arrhythmias (OR, 4.659; 95% CI, 1.481–14.653; *p* = 0.008) were also associated with death of 3 months. Our analysis showed that safety measures undertaken in this hospital, including screening of COVID-19 infection and use of personal protection equipment for conducting PCI did not compromise the surgical outcome as compared with PCI under normal condition, although there were slight increases in incidence of malignant arrhythmia and Takotsubo Syndrome.

## Introduction

The novel Coronavirus Disease-2019 (COVID-19) spread rapidly in China after December 2019^1^. As of September 5, 2020, 85,122 confirmed cases were reported in China. In addition, a total of 818,580 close contacts were traced during last 10 months and 6110 close contacts are still under medical observation. Because of the strong infectivity of the SARS-nCov-2 that causes COVID-19, the operation and treatment in many medical institutions were affected^2^. During this time, elective percutaneous coronary intervention (PCI) was cancelled or postponed in the hospital and only emergency PCI was allowed to proceed. At the beginning of the outbreak of COVID-19, information regarding the source and mode of transmission of the virus was not clear. Therefore, Chinese Medical Association released expert consensus on operation protocols for chest pain center during the COVID-19 pandemic, for suspected or confirmed COVID-19 patients, thrombolytic therapy is recommended for those without thrombolytic contraindications and interventional therapy should be used applied only after assessing the risk and benefit of thrombolytic therapy. Therefore, during this period, some emergency PCI were changed to thrombolytic therapy, some PCI centers delayed or canceled the emergency PCI operation. In the next few months, after the virus transmission through the respiratory tract and droplets were identified, new guidelines for catheterization laboratories were published^3^ and emergency PCI were resumed in all centers.

In order to ensure safety of patients and medical staff, our hospital applied relevant procedures for admission and surgical management for PCI patients with acute myocardial infarction (AMI).

The study was conducted to compare the therapeutic outcomes of patients who underwent PCI under the safety procedure in 2020 and normal condition in 2019, and the findings would help identify and improve factors and measures that enable effective treatment of AMI patients in the COVID-19 pandemic.

## Subject and methods

### Subjects

AMI patients who received emergency PCI between January 24 and April 30, 2019 and 2020 were included in this retrospective study as 2019 and COVID-19 groups. Patients were included and excluded based on 2018 ESC/EACTS Guidelines on Myocardial Revascularization^4^. Patients were included if the onset was < 12 h but had ST-segment elevation, or the onset was > 12 h with persistent symptom or instable haemodynamics and malignant arrhythmia, or no ST-segment elevation, but had evidence of myocardial infarction-derived ischaemia with at least one of the following conditions: haemodynamic instability, cardiogenic shock, ongoing chest pain, life-threatening arrhythmias or cardiac arrest, mechanical complications of myocardial infarction, heart failure, recurrent dynamic ST-segment or T-wave changes, particularly with intermittent ST-segment elevation. In addition, patients with symptom onset > 12 h, pain free and with stable haemodynamics were excluded.

All patients received 300 mg aspirin and 180 mg ticagrelor before operation. Demographic and clinical data of patients, such as door-to-balloon (DTB) time, operation time and complications were retrieved from hospital medical databases. Patients were diagnosed AMI based on electrocardiogram (ECG) and/or cardiac enzyme profiles using the fourth universal definition of myocardial infarction^5^. The primary outcomes were 3-month all-cause mortality, re-hospitalization for heart failure and re-hospitalization for acute coronary syndrome. In-hospital outcomes were death from any reason, heart failure, malignant arrhythmias and cardiogenic shock. Other outcome measures included length of hospital stay, DTB time and operation time.

### Safety measures for COVID-19

Based on expert consensus^6^ and other guidelines such as World Health Organization guidelines (https://www.who.int/publications/i/item/10665-331495), four personal protective levels were set up: general protection with surgical masks, level I protection requiring to wear work clothes, work caps, surgical masks and gloves, level II protection requiring to wear work clothes, work caps, isolation gowns, shoe covers, medical protective masks, goggles or protective face screen and level III protection requiring to wear N95 masks and double layer gloves in addition to level II protections. According to exposure risks, the hospital was divided into three areas that implemented different levels of protection. “Red area” was designated for high-risk areas such as fever clinic, emergency department, laboratory, pathology department, intensive care unit and other departments that might be directly in contact with patients who had not been tested for SARS-nCov-2 nucleic acid. The protection for the red area was level III. “Yellow area" was designed as a buffer area for coronary care unit (CCU). The yellow area ward was a separate nursing unit in the hospital, and each room only accommodated one emergency patient. The protection in the yellow area was “level II”. “Green area” was designated for general cardiology ward. Patients were transferred to the area after nucleic acid test was done, the result was negative and the isolation period was over. For this area, the protection was level I.

### Primary PCI procedure for COVID-19 group

**Figure 1:**
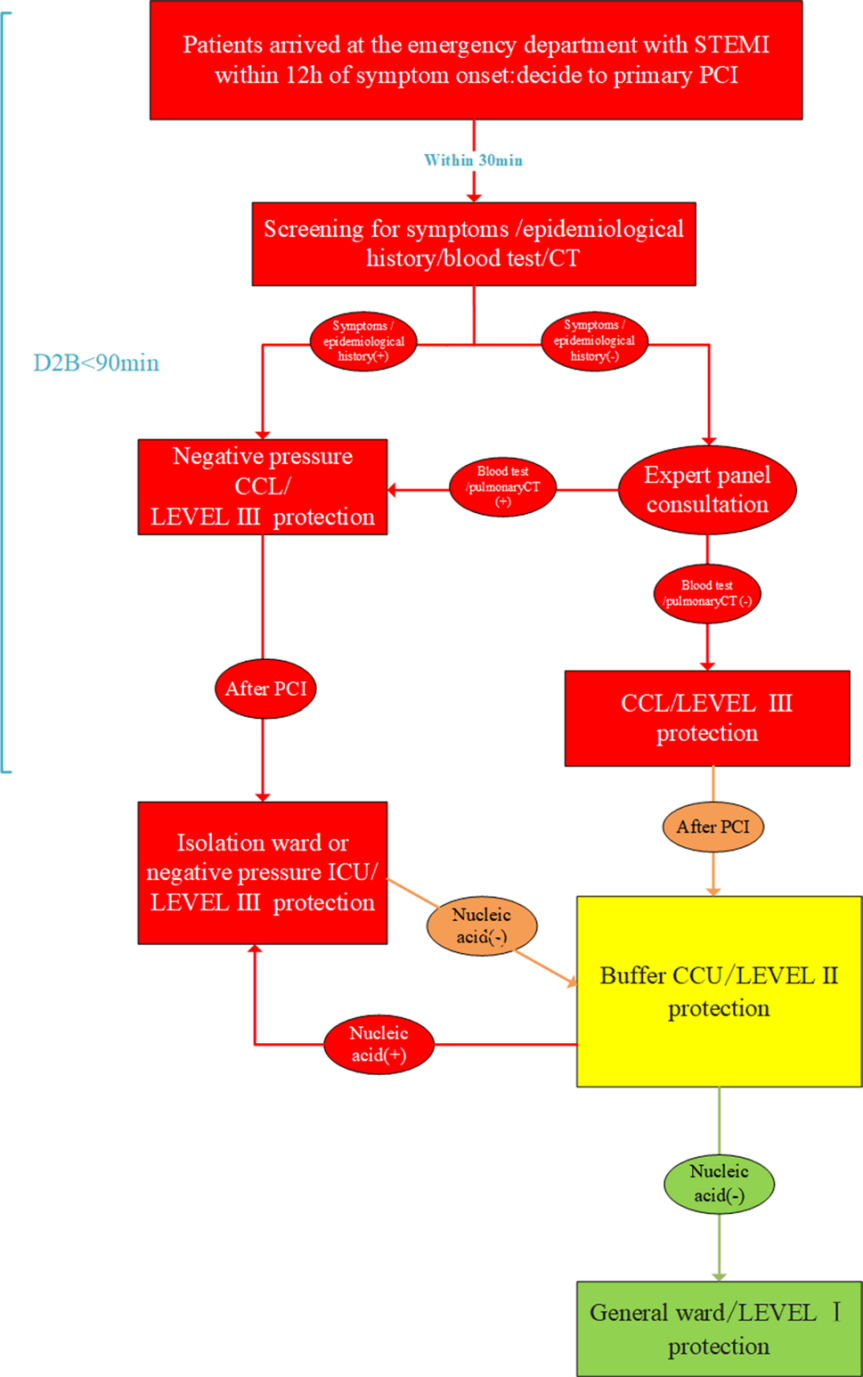
Safety measures and procedures for primary PCI in AMI patients in 2020 coronavirus disease (COVID-19) pandemic period at this hospital.

While patients in 2019 group were treated as usual, the patients in COVID-19 group were requested to wear a surgical mask in all areas as general protection. Within 30 min after arriving at emergency department, patients were examined for epidemiological history, symptoms such as fever and cough, chest CT and routine blood tests. Patients with epidemiological history and symptoms such as fever and cough were routed to negative pressure cardiac catheterization laboratory (CCL) for PCI. During PCI, the results of chest CT and routine blood tests were assessed by a five-person expert panel to determine if the patients from regular CCL should be sent to yellow area or to negative pressure CCL. Patients routed to isolation ward or negative pressure ICU were transferred to the buffer ward after medical isolation observation (usually for a week) and negative nucleic acid detection^7^. Other patients (without symptoms and epidemiological history) were sent to regular CCL for PCI after consultation with COVID-19 expert panel (composed of respiratory department, radiology department, intensive care unit, infection department and emergency department). After the operation, the patients were transferred to the buffer ward (yellow area) for COVID-19 test and isolation observation. After coronavirus infection was completely excluded, patients were allowed to stay in the green area in department of cardiology (Fig. 1). All methods were performed in accordance with the relevant guidelines and regulations.

### Statistical analysis

The normality of distribution of continuous variables was tested by one-sample Kolmogorov–Smirnov test. Continuous variables with normal distribution were presented as mean ± standard deviation and analyzed using Student’s *t* test. Categorical variables are presented as percentage, and analyzed using χ^2^ test Fisher's exact test, when appropriate. Multivariate Cox regression was performed to analyze the independent risk factors for MACE and death. Odds ratio was calculated using COX regression. Variables in the adjusted model were age (≥ 75), gender, BMI (≥ 30), smoking, DTB, operation time, hospital stay, DES (drug eluting stent), IABP use, final TIMI-3, and comorbidities which include diabetes, hypertension, hyperlipidemia, Takotsubo syndrome, cardiogenic shock, malignant arrhythmias, heart failure and grouping (2019 group vs COVID-19 group). A value of *p* < 0.05 was considered significant. SPSS version 25.0 for Windows (SPSS Inc., Chicago, IL, USA) was used to analysis all data.

### Ethics approval

This study was approved by the Ethical Committee of Capital Medical University, China. Written and informed consents were obtained from all patients.

## Results

For PCI Division, a total of 37 patients were classified between January 24 and April 30, 2020, and 4 and 33 patients were routed to red and yellow zones, respectively, but none of them was confirmed positive; a total of 95 patients were stratified between May 1 and December 30, 2020, 13 and 82 patients were routed to red and yellow zones, respectively, among them two patients in red zone were confirmed positive.

**Figure 2:**
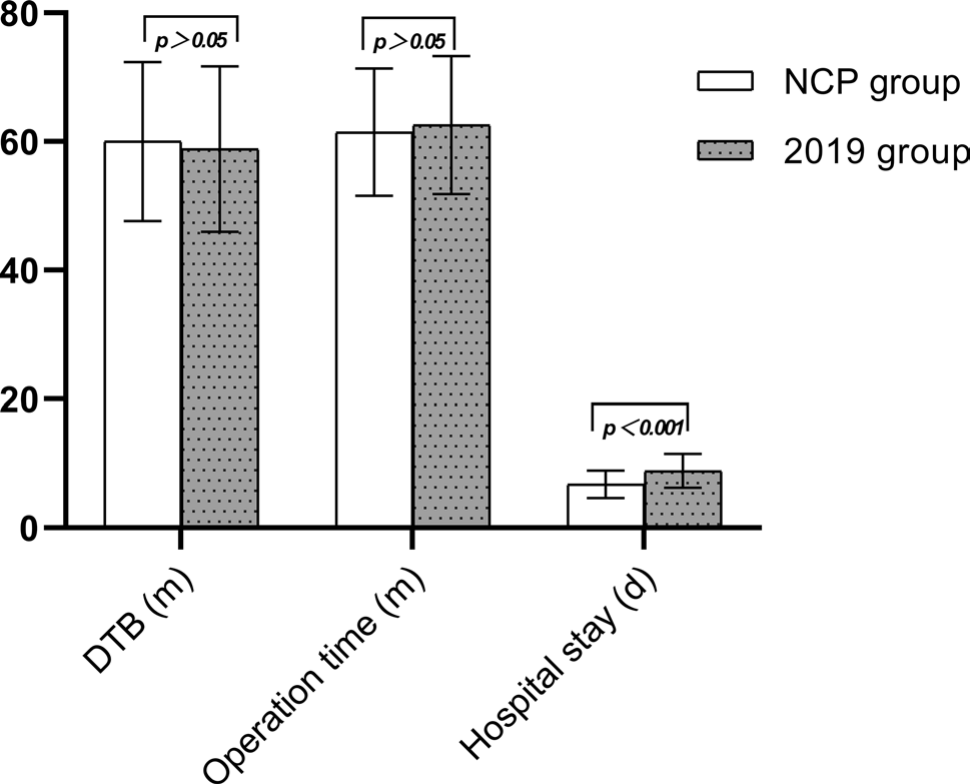
Comparison of DTB, operation time and hospital stay of AMI patients undergoing PCI in 2020 coronavirus disease (COVID-19) pandemic period and 2019.

**Figure 3:**
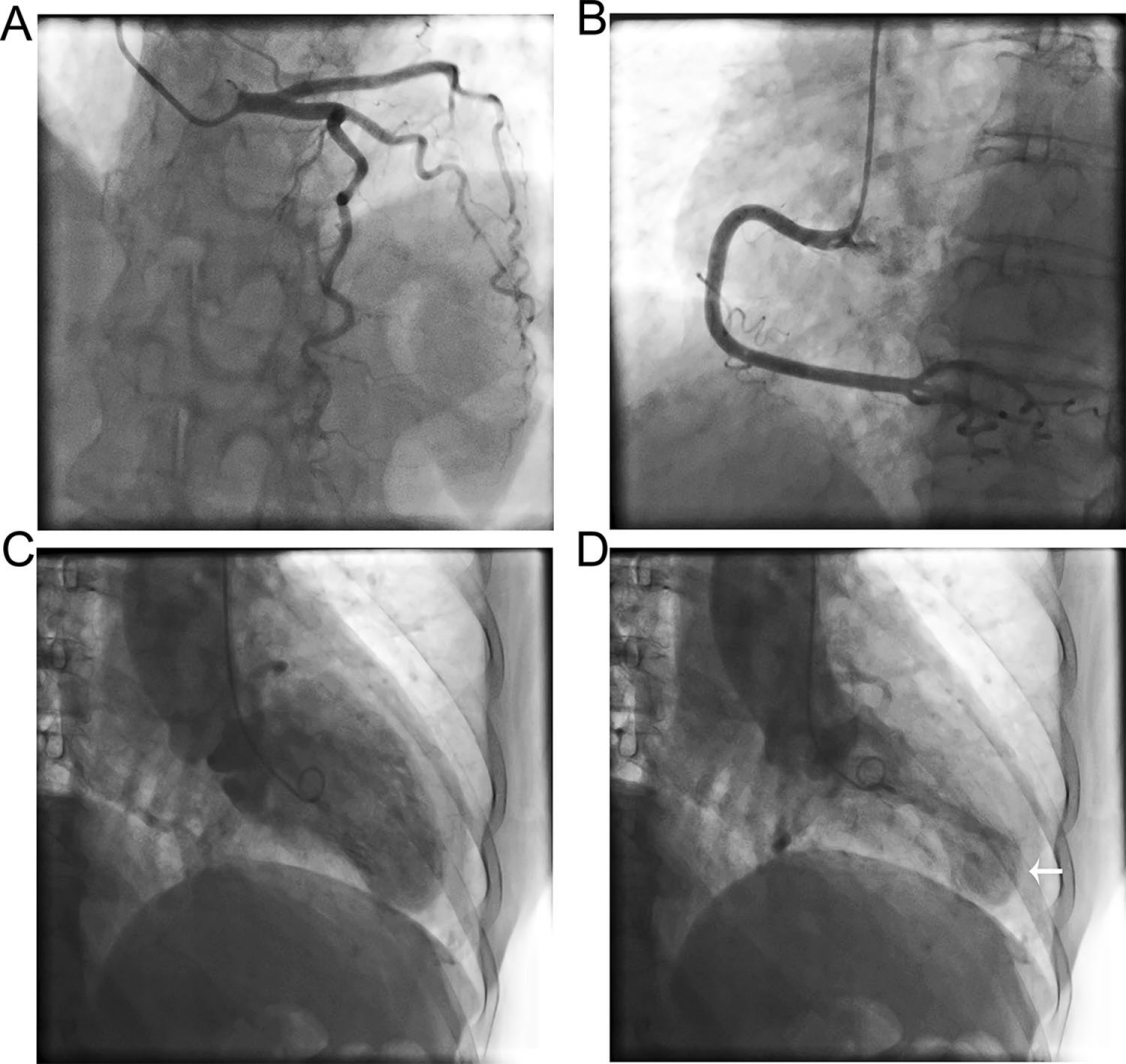
Coronary angiography and left ventriculography of a 81-year old woman showing apical TTS. (**A**) Left coronary artery; (**B**) right coronary artery; (**C**) diastolic period; (**D**) systolic period. Arrow points to TTS.

**Figure 4:**
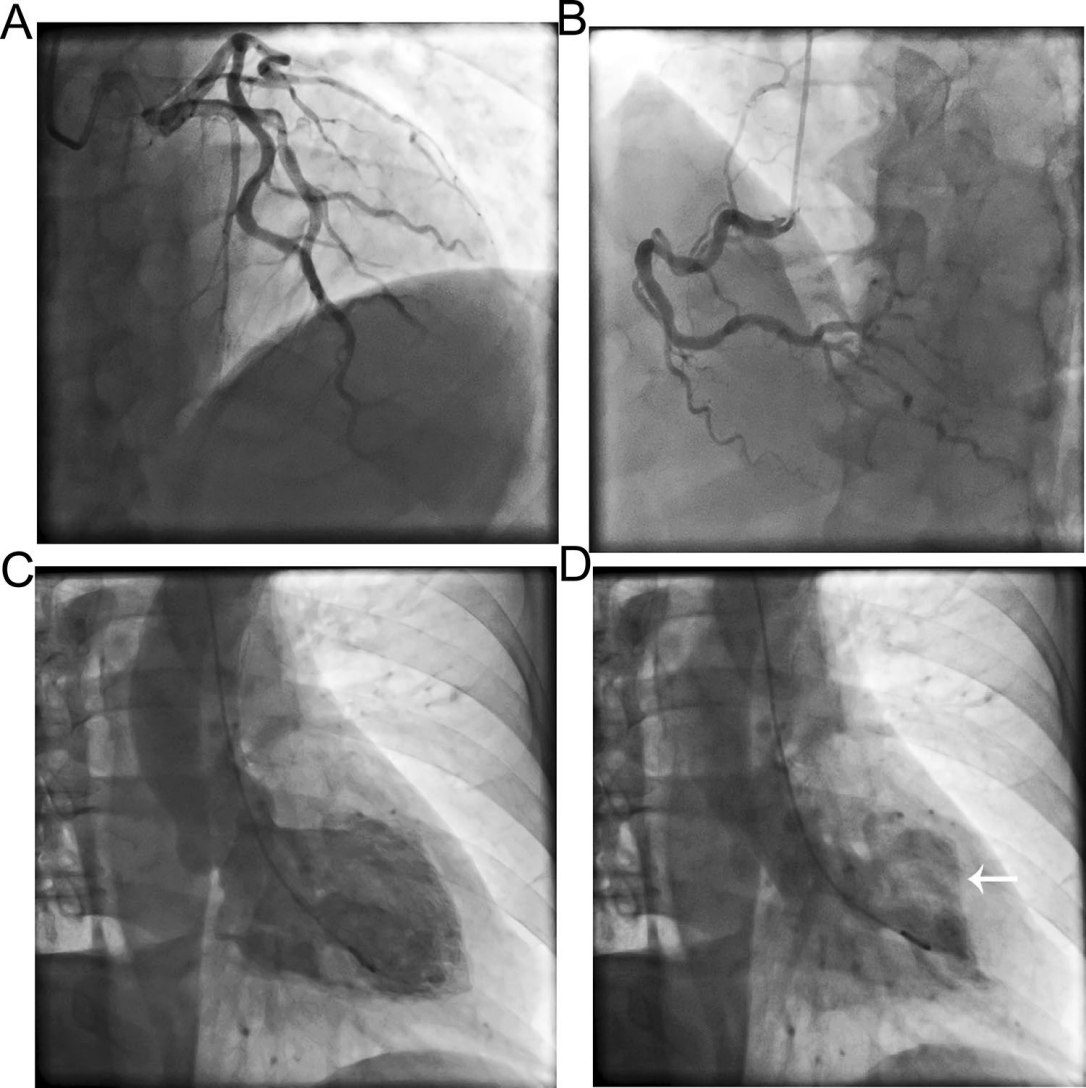
Coronary angiography and left ventriculography of a 55-year old man showing focal TTS. (**A**) Left coronary artery; (**B**) right coronary artery; (**C**) diastolic period; (**D**) systolic period. Arrow points to TTS.

**Figure 5:**
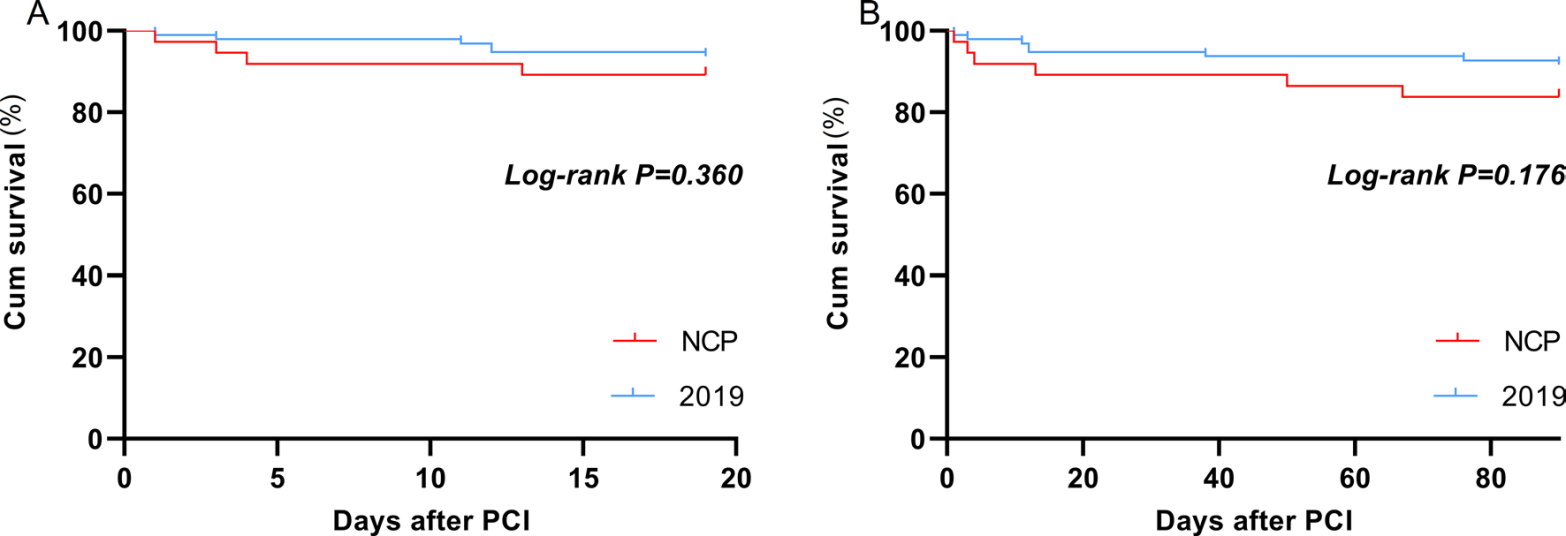
Kaplan–Meier curves for death in hospital (**A**) and 3-month follow-up (**B**) of AMI patients undergoing PCI in 2020 coronavirus disease (COVID-19) pandemic period and 2019.

A total of 37 and 96 AMI patients undergoing primary PCI between January 24 and April 30, 2020 and 2019 were included in this study (Table 1). The age and BMI in COVID-19 group and 2019 group were 59.70 ± 13.76 vs. 58.60 ± 11.19, and 26.64 ± 4.68 vs. 25.63 ± 4.16, respectively. There was no significant difference in age, gender, BMI and comorbidity between the two groups. There was no significant difference in hypertension, diabetes, hyperlipidemia and smoking between the two groups. Drug eluting stent (DES) were implanted in 27 (72.97%) patients in COVID-19 group and 79 (79.17%) patients in 2019 group (*p* > 0.05). Six (16.22%) patients in COVID-19 group and 19 (19.76%) patients in 2019 group received percutaneous transluminal coronary angioplasty (PTCA, *p* > 0.05). DTB time and operation time of COVID-19 and 2019 groups were similar (60.0 ± 12.39 vs 58.83 ± 12.85 m, *p* = 0.636; 61.46 ± 9.91 vs 62.55 ± 10.72 m, *p* = 0.592) (Fig. 2). There was no significant difference in the use of IABP between the two groups (10.81% vs 7.29%, *p* = 0.509). Thirty-three patients in the COVID-19 group and 96 patients in the 2019 group reached TIMI-3 blood flow. There was no significant difference between the two groups (89.19% vs 94.79%, *p* = 0.249). There was no significant difference in contrast medium dose, X-ray time, and X-ray dose between the two groups (123.03 ± 20.28 vs 127.30 ± 22.41 ml, *p* = 0.314; 23.03 ± 4.16 vs 24.32 ± 5.85 m, *p* = 0.220; 1514.54 ± 166.96 vs 1561.58 ± 195.67 mGy, *p* = 0.199). Four patients in COVID-19 group were diagnosed as having Takotsubo Syndrome (TTS). The incidence was significantly higher than that in the 2019 group (10.81% vs 1.04%, *p* = 0.008). Three of them were women aged 60–81 with apical TTS, and the other was man aged 55 with focal TTS (Figs. 3 and 4). However, coronary angiography showed no severe stenosis in all 4 patients. The hospital stay time in COVID-19 group was significantly shorter than in 2019 group (6.78 ± 2.14 vs 8.85 ± 2.64 days, *p* < 0.001) (Fig. 2). There was no significant difference between the two groups in incidence of cardiogenic shock, heart failure and death during hospital (Fig. 5). However, the incidence of malignant arrhythmia was significantly higher than in COVID-19 group than 2019 group (16.22% vs 5.21%, *p* = 0.039).

**Table 1:** Patient characteristics.

	NCP group (n = 37)	2019 group (n = 96)	T/χ^2^	*p*
Age, y	59.70 ± 13.76	58.60 ± 11.19	0.475	0.636
BMI, kg/m^2^	26.64 ± 4.68	25.63 ± 4.16	1.215	0.227
Male, n (%)	26 (70.27)	71 (73.96)	0.184	0.668
Hypertension, n (%)	23 (62.16)	58 (60.42)	0.034	0.853
Diabetes, n (%)	19 (51.35)	49 (52.04)	0.001	0.974
Hyperlipidemia, n (%)	25 (67.57)	53 (55.21)	1.682	0.195
Smoke, n (%)	21 (56.76)	56 (58.33)	0.027	0.869
DTB, min	60.0 ± 12.39	58.83 ± 12.85	0.474	0.636
Operation time, min	61.46 ± 9.91	62.55 ± 10.72	− 0.538	0.592
Drug eluting stent, n (%)	27 (72.97)	76 (79.17)	0.151	0.698
Stent number	1.05 ± 0.82	1.10 ± 0.80	− 0.322	0.748
PTCA, n (%)	6 (16.22)	19 (19.76)	0.224	0.636
IABP, n (%)	4(10.81)	7(7.29)	0.436	0.509
Final TIMI-3, n (%)	33(89.19)	91(94.79)	1.329	0.249
X-ray time, min	23.03 ± 4.16	24.32 ± 5.85	− 1.232	0.220
X-ray dose, mGy	1514.54 ± 166.96	1561.58 ± 195.67	− 1.292	0.199
Contrast medium dose, ml	123.03 ± 20.28	127.30 ± 22.41	− 1.011	0.314
Radial artery approach, n (%)	28(75.68)	85(94.79)	2.896	0.089
Puncture complications, n (%)	2(5.4%)	4(4.17%)		0.670
Takotsubo Syndrome, n (%)	4 (10.81)	1 (1.04)	7.045	0.008
Hospital-stay, day	6.78 ± 2.14	8.85 ± 2.64	− 4.255	< 0.001
Cardiogenic shock, n (%)	3 (8.11)	4 (4.17)	0.832	0.362
Malignant arrhythmias, n (%)	6 (16.22)	5 (5.21)	4.266	0.039
Heart failure, n (%)	2 (5.41)	10 (10.42)	0.817	0.366
Death, n (%)	4 (10.81)	6 (6.25)	0.799	0.371

The patients were followed up for 3 months. During the follow-up, there was no difference in re-hospitalization rates due to heart failure and acute coronary syndrome (ACS) and mortality between the two group (Fig. 5). The incidence of major adverse cardiac events (MACE) was also not statistically different between the two groups (Table 2). Additionally, multivariable COX regression analyses demonstrated that cardiogenic shock (OR, 11.53; 95% CI, 2.888–46.036; *p* = 0.001), malignant arrhythmias (OR, 7.176; 95% CI, 1.893–27.203; *p* = 0.004) and age ≥ 75 years (OR, 6.718; 95% CI, 1.738–25.964; *p* = 0.006) were associated with MACE (Table 3), while grouping, gender, BMI (≥ 30), smoking, DTB, operation time, DES, IABP use, final TIMI-3, comorbidities and group were not significant to enter into the final regression model (*p* > 0.05, Table 3). Cardiogenic shock (OR, 17.663; 95% CI, 5.5–56.762; *p* < 0.001) and malignant arrhythmias (OR, 4.659; 95% CI, 1.481–14.653; *p* = 0.008) were also associated with death of 3 months (Table 3).

**Table 2:** Major adverse cardiac events in 3-month follow-up.

	NCP group (n = 37)	2019 group (n = 96)	χ^2^	*p*
Major adverse cardiac events, n (%)	10 (35.13)	14 (14.58)	2.796	0.094
Re-hospitalization for heart failure, n (%)	3 (8.11)	4 (4.17)	0.832	0.362
Re-hospitalization for ACS, n (%)	1 (2.7)	2 (2.08)		1.000
Death, n (%)	6 (16.22)	8 (8.33)	1.762	0.184

**Table 3:** Multivariate COX analysis for MACE and death in 3 months after PCI.

Variable	β	SE	Wald X^2^	OR	p	95% CI	
**MACE**							
Cardiogenic shock	1.790	0.581	9.485	5.988	0.002	1.917	18.702
Malignant arrhythmias	1.601	0.466	11.807	4.959	0.001	1.989	12.362
Age ≥ 75 years	1.126	0.492	5.238	3.083	0.022	1.175	8.084
**Death**							
Cardiogenic shock	2.871	0.595	23.266	17.663	< 0.001	5.500	56.726
Malignant arrhythmias	1.539	0.585	6.929	4.659	0.008	1.481	14.653

Variables in adjusted model were age (≥ 75), gender, BMI (≥ 30), smoking, DTB, operation time, hospital stay, DES (drug eluting stent), IABP use, final TIMI-3, diabetes, hypertension, hyperlipidemia, Takotsubo syndrome, cardiogenic shock, malignant arrhythmias, heart failure and grouping (2019 group vs COVID-19 group).

## Discussion

Our study shows that emergency PCI treatment can be safely performed when protective measures are well established to avoid the COVID-19 transmission and the therapeutic outcomes are comparable to PCI conducted in non-COVID-19 condition. According to the new coronavirus China guidelines^8^, this hospital developed safety measures and procedures for COVID-19 such as zoned areas and different levels of protection. With these measures and procedures, AMI patients were timely treated with PCI even during the COVID-19 pandemic. In the procedures the risk level was determined based on information and experience from Wuhan and the virus was believed to mainly transmitted by droplets, feces, urine, and even aerosols^9^. Considering the closed environment of the CCL and the uncertainty of whether the patients are infected, the procedures required that the PCI team uses level III protection and the CCL was designated as red area for the highest safety. PCI patients with epidemiological history and respiratory symptoms such as fever and asthma are allocated to isolation ward or negative pressure ICU after operation. No patients were not allowed to gain direct access to the green area from the red area to prevent potential infection. In addition, patients were examined using blood tests and pulmonary CT as a fast way to rule out the possibility of infection. To implement the procedures, a team of experienced experts were in place 24 h a day, seven days a week to interpret the epidemiological data, pulmonary CT and routine blood tests to identify suspected COVID-19 infection. The experts team decided which patients (without epidemiological history and respiratory symptoms) need to use the negative pressure CCL, and be allocated to isolation ward or negative pressure ICU after operation. Other patients will be operated in the regular CCL, and stay in buffer CCU until COVID-19 nucleic acid test was negative. Avoid waiting for nucleic acid test results shorten the waiting time for emergency PCI. The identification of patients by expert team is crucial for protection procedure. As a result of safety measures, patients in PCI and other department were routed into zones of different risk levels for management as shown in Fig. 1 and reported previously^10^, for better infection control and CCL use. As a consequence of the collective efforts, no patients and hospital staff in the cardiology department are infected with COVID-19. To assess the possible impact of the safety measures on the outcomes of PCI, we compared the clinical outcomes of COVID-19 group with 2019 group, which was performed under normal condition. Compared with 2019, only emergency and fever patients came to the hospital for treatment in 2020, leading to fewer patients. Although these patients were subjected to a series of additional screening and examinations prior to the surgery as compared with 2019, the DTB time was similar between the two groups. This might be due to reduced number of patients, which allowed faster handling and treatment by the medical team. Also, it indicated that wearing level III protection may not affect the operation time. Wearing protective clothing and multi-layers of gloves are often uncomfortable. However, most of the emergency PCI physicians are well trained and skillful. Such inconvenience did not impact the stent implantation for rapid recanalization of infarct-related arteries. As a result, compared with previous year, there was no significant increase in puncture complications, the use of X radiation and contrast agents. The hospitalization time of patients in COVID-19 group was significantly shorter than 2019 group. This may be because during the COVID-19, patients were encouraged to leave the hospital as soon as possible to avoid possible infection risk of the virus. During the 3-month follow-up, there was no significant difference in readmission rate and mortality between the two groups. However, it is worth noting that the incidence of malignant arrhythmia was somewhat higher in COVID-19 group, which could post potential risk to patients. Multivariable COX regression showed that malignant arrhythmias is an independent risk factor for MACE and death of 3 months. Malignant arrhythmia may be related to sympathetic excitation caused by tension, but it is unclear why its incidence is higher in the COVID-19 group. Available data suggest that AMI patients with early VT (ventricular tachycardia)/VF (ventricular fibrillation) have increased 30-day mortality^11^. Beta-blockers, such as amiodarone and lidocaine, should be considered and repetitive electrical cardioversion or defibrillation may be necessary^12^. In addition, cardiogenic shock is also an important independent risk factor for MACE and death of 3 months. Cardiogenic shock is a leading cause of death, with in-hospital mortality rates ≥ 50%^13^. The most important treatment for cardiogenic shock is immediate reperfusion and total revascularization if there is multivessel disease. IABP and other mechanical left ventricle assist devices (LVADs) are often used in patients with cardiogenic shock. However, evidence regarding the benefits of IABP and LVADs are limited^14,15^. DTB is considered as an important factor affecting the prognosis of acute myocardial infarction^16^. However, in our study, DTB is not an independent risk factor of MACE probable because all patients in this study completed emergency PCI within 90 min. In the study, grouping was not found to be an independent risk factor of MACE. This is likely because none of patient undergoing PCI in both COVID-19 group and 2019 group was infected by COVID-19, because active protective and screening measures were taken during the epidemic period. As a consequence, the severity of the patient's myocardial infarction might not have affected by COVID-19. However, since only 37 patients were involved in this study in COVID-19 group, the impact of COVID-19 on MACE in PCI needed to be further assessed in large sample study to drawn a more solid conclusion. Age ≥ 75 was also a risk factor for MACE in the present study, which might be attributed to their comorbidities, higher risk of bleeding and decreased renal function. On other hand, individual comorbidity may not be independent risk factor of MACE. It is important to use specific strategies to reduce bleeding risk and apply antithrombotic therapies with primary PCI^17^. Another finding is that the incidence of TTS is higher in the COVID-19 group. TTS is estimated to represent approximately 1–3%^18,19^ of all and 5–6%^20^ of female patients presenting with suspected STEMI. Sympathetic stimulation is considered one of the major pathophysiological mechanisms of TTS^21^. The novel coronavirus pneumonia is stressful, which would make people panic and trigger dramatic emotional change. This may attribute to the increased incidence of TTS. Catecholamine storm leads to malignant arrhythmia in TTS^22^ and AMI^23^. Therefore, beta blockers may be considered to treat malignant arrhythmia during this period.

Ischemic heart disease is the single most common cause of death in the world. Primary PCI is the preferred reperfusion strategy in patients with STEMI within 12 h of symptom onset. For better outcomes, DTB time should be controlled within 90 min^24^. The mortality in STEMI patients is influenced by many factors. Patients with cardiovascular disease who developed COVID-19 may have a higher risk of mortality^25^. This is similar to the situation with acute respiratory syndrome coronavirus (SARS-CoV)^26^ and the Middle East respiratory syndrome coronavirus (MERS-CoV)^27^. How COVID-19 is associated with cardiovascular (CV) injury is not clear. Possible mechanisms include viral myocarditis, ACE-2 receptor-mediated CV injury, microvascular dysfunction and cytokine release syndrome^28,29^. ACC/AHA Management of AMI During the COVID-19 Pandemic suggests that primary PCI remains as standard of care for STEMI patients at PCI capable hospitals when it can be provided in a timely fashion, with an expert team outfitted with PPE in a dedicated CCL room^30^. Our experience demonstrates that primary PCI can be performed during the COVID-19 pandemic to obtain therapeutic outcomes comparable to these obtained in normal condition.

Because COVID-19 is infectious in the latent period, and are more infectious within 5 days after the onset of the disease^31^, and patients cannot be excluded for infection in a short time, it is important to separate infected patients from uninfected patients in the treatment process. AMI patients often suffer from severe chest pain and are at risk of hemodynamic collapse, and it is difficult for them to wait for COVID-19 test result even from rapid nucleic acid assay. Therefore, the clinical conditions of patients should be assessed safely and rapidly to allow timely treatment.

There are limitations in our study. First of all, due to the pandemic situation, the number of AMI patients was small, which may lead to bias in the results. Secondly, the follow-up time was short. Finally, due to the nature of the pandemic, the severity of infection in Beijing was not able to compare with other regions, which may impact the representativeness of our study.

## Conclusion

Four levels of personal protection and three protective zones were set up our hospital during the COVID-19 epidemic period to minimize virus transmission. These measures in cope with comprehensive virus screen protocols resulted in no COVID-19 infection in the patients and medical staff in the cardiology department, and allowed timely implementation of primary PCI for AMI patients. Compared with PCI performed in 2019, PCI performed in COVID-19 group with the safety measures and procedures did not increase DTB time and had similar primary outcomes (death and MACE) in 3 months followed up, although the incidences of malignant arrhythmia and TTS were higher. Cardiogenic shock and malignant arrhythmias was also associated with death of 3 months. MACE in PCI was found to be significantly associated with cardiogenic shock, malignant arrhythmias and advanced age (≥ 75 years). Since this study is a single center retrospective, the sample size is small, the follow-up time is short, the conclusions need to be validated in large multicenter studies.

## Data Availability

The datasets used and/or analyzed during the current study are available from the corresponding author on reasonable request.

## References

[1] Guan WJ (2020). Clinical characteristics of coronavirus disease 2019 in China. N. Engl. J. Med..

[2] Zhao XY (2020). Clinical characteristics of patients with 2019 coronavirus disease in a non-Wuhan area of Hubei Province, China: A retrospective study. BMC Infect. Dis..

[3] Ma X, Liu Y, Fu J, Xu B (2020). Management strategy and recommendations for catheterization laboratories during outbreak of coronavirus disease 2019. Chin. Circ. J..

[4] Neumann FJ (2019). 2018 ESC/EACTS guidelines on myocardial revascularization. EuroIntervention.

[5] Thygesen K (2018). Fourth universal definition of myocardial infarction (2018). Circulation.

[6] Li G (2020). Expert consensus on personal protection in different regional posts of medical institutions during COVID-19 pandemic period. Chin. J. Infect. Control.

[7] Yuan B (2020). Recurrence of positive SARS-CoV-2 viral RNA in recovered COVID-19 patients during medical isolation observation. Sci. Rep..

[8] National Health Commission, National Administration of Traditional Chinese Medicine (2020). Novel coronavirus pneumonia diagnosis and treatment guideline. J. Infect. Dis..

[9] Zou L (2020). SARS-CoV-2 viral load in upper respiratory specimens of infected patients. N. Engl. J. Med..

[10] Liu Y, Wang M, Shen Y, Chen J (2020). Analysis of operation procedure and effect for emergency surgery in general hospital during novel coronavirus pneumonia period. BMC Surg..

[11] Roolvink V (2016). Early intravenous beta-blockers in patients with ST-segment elevation myocardial infarction before primary percutaneous coronary intervention. J. Am. Coll. Cardiol..

[12] Piccini JP (2011). Antiarrhythmic drug therapy for sustained ventricular arrhythmias complicating acute myocardial infarction. Crit. Care Med..

[13] Goldberg RJ, Spencer FA, Gore JM, Lessard D, Yarzebski J (2009). Thirty-year trends (1975 to 2005) in the magnitude of, management of, and hospital death rates associated with cardiogenic shock in patients with acute myocardial infarction: A population-based perspective. Circulation.

[14] Starling RC (2011). Results of the post-U.S. Food and Drug Administration-approval study with a continuous flow left ventricular assist device as a bridge to heart transplantation: A prospective study using the INTERMACS (Interagency Registry for Mechanically Assisted Circulatory Support). J. Am. Coll. Cardiol..

[15] Thiele H (2012). Intraaortic balloon support for myocardial infarction with cardiogenic shock. N. Engl. J. Med..

[16] Terkelsen CJ (2011). Health care system delay and heart failure in patients with ST-segment elevation myocardial infarction treated with primary percutaneous coronary intervention: Follow-up of population-based medical registry data. Ann. Intern. Med..

[17] Bueno H (2011). Primary angioplasty vs. fibrinolysis in very old patients with acute myocardial infarction: TRIANA (TRatamiento del Infarto Agudo de miocardio eN Ancianos) randomized trial and pooled analysis with previous studies. Eur. Heart J..

[18] Prasad A (2014). Incidence and angiographic characteristics of patients with apical ballooning syndrome (takotsubo/stress cardiomyopathy) in the HORIZONS-AMI trial: An analysis from a multicenter, international study of ST-elevation myocardial infarction. Catheter Cardiovasc. Interv..

[19] Bybee KA (2004). Clinical characteristics and thrombolysis in myocardial infarction frame counts in women with transient left ventricular apical ballooning syndrome. Am. J. Cardiol..

[20] Redfors B (2015). Mortality in Takotsubo syndrome is similar to mortality in myocardial infarction—A report from the SWEDEHEART registry. Int. J. Cardiol..

[21] Templin C (2015). Clinical features and outcomes of Takotsubo (stress) cardiomyopathy. N. Engl. J. Med..

[22] Ohkubo K (2011). Functional atrioventricular conduction block in an elderly patient with acquired long QT syndrome: Elucidation of the mechanism of block. J. Electrocardiol..

[23] Enjoji Y (2009). Catheter ablation of fatal ventricular tachyarrhythmias storm in acute coronary syndrome—Role of Purkinje fiber network. J. Interv. Card. Electrophysiol..

[24] Ibanez B (2017). ESC Guidelines for the management of acute myocardial infarction in patients presenting with ST-segment elevation. Rev. Esp. Cardiol. (Engl. Ed.).

[25] Ganatra S (2020). Management of cardiovascular disease during coronavirus disease (COVID-19) pandemic. Trends Cardiovasc. Med..

[26] Chan JW (2003). Short term outcome and risk factors for adverse clinical outcomes in adults with severe acute respiratory syndrome (SARS). Thorax.

[27] Badawi A, Ryoo SG (2016). Prevalence of comorbidities in the Middle East respiratory syndrome coronavirus (MERS-CoV): A systematic review and meta-analysis. Int. J. Infect. Dis..

[28] Zheng YY, Ma YT, Zhang JY, Xie X (2020). COVID-19 and the cardiovascular system. Nat. Rev. Cardiol..

[29] Nicin L (2020). Cell type-specific expression of the putative SARS-CoV-2 receptor ACE2 in human hearts. Eur. Heart J..

[30] Mahmud, E. *et al.* Management of acute myocardial infarction during the COVID-19 pandemic. *J. Am. Coll. Cardiol.* **96**, 336–34510.1016/j.jacc.2020.04.039 (2020).

[31] Lauer SA (2020). The incubation period of coronavirus disease 2019 (COVID-19) from publicly reported confirmed cases: Estimation and application. Ann. Intern. Med..

